# An Artificial Intelligence–Based Smartphone App for Assessing the Risk of Opioid Misuse in Working Populations Using Synthetic Data: Pilot Development Study

**DOI:** 10.2196/45434

**Published:** 2023-05-30

**Authors:** A B M Rezbaul Islam, Khalid M Khan, Amanda Scarbrough, Mariah Jade Zimpfer, Navya Makkena, Adebola Omogunwa, Sheikh Iqbal Ahamed

**Affiliations:** 1 Department of Computer Science Sam Houston State University Huntsville, TX United States; 2 Department of Public Health Sam Houston State University Huntsville, TX United States; 3 Department of Computer Science Marquette University Milwaukee, WI United States

**Keywords:** opioid overused disorder, OUD, mobile health, mHealth, artificial intelligence, smartphone app, opioids, application, caregivers, mobile app

## Abstract

**Background:**

Opioid use disorder (OUD) is an addiction crisis in the United States. As recent as 2019, more than 10 million people have misused or abused prescription opioids, making OUD one of the leading causes of accidental death in the United States. Workforces that are physically demanding and laborious in the transportation, construction and extraction, and health care industries are prime targets for OUD due to high-risk occupational activities. Because of this high prevalence of OUD among working populations in the United States, elevated workers’ compensation and health insurance costs, absenteeism, and declined productivity in workplaces have been reported.

**Objective:**

With the emergence of new smartphone technologies, health interventions can be widely used outside clinical settings via mobile health tools. The major objective of our pilot study was to develop a smartphone app that can track work-related risk factors leading to OUD with a specific focus on high-risk occupational groups. We used synthetic data analyzed by applying a machine learning algorithm to accomplish our objective.

**Methods:**

To make the OUD assessment process more convenient and to motivate potential patients with OUD, we developed a smartphone-based app through a step-by-step process. First, an extensive literature survey was conducted to list a set of critical risk assessment questions that can capture high-risk behaviors leading to OUD. Next, a review panel short-listed 15 questions after careful evaluation with specific emphasis on physically demanding workforces—9 questions had two, 5 questions had five, and 1 question had three response options. Instead of human participant data, synthetic data were used as user responses. Finally, an artificial intelligence algorithm, naive Bayes, was used to predict the OUD risk, trained with the synthetic data collected.

**Results:**

The smartphone app we have developed is functional as tested with synthetic data. Using the naive Bayes algorithm on collected synthetic data, we successfully predicted the risk of OUD. This would eventually create a platform to test the functionality of the app further using human participant data.

**Conclusions:**

The use of mobile health techniques, such as our mobile app, is highly promising in predicting and offering mitigation plans for disease detection and prevention. Using a naive Bayes algorithm model along with a representational state transfer (REST) application programming interface and cloud-based data encryption storage, respondents can guarantee their privacy and accuracy in estimating their risk. Our app offers a tailored mitigation strategy for specific workforces (eg, transportation and health care workers) that are most impacted by OUD. Despite the limitations of the study, we have developed a robust methodology and believe that our app has the potential to help reduce the opioid crisis.

## Introduction

Opioid use disorder (OUD) is a pressing health challenge that has been cited as an “overwhelming public health problem in the United States” [1]. As recent as 2019, more than 10 million people in the United States have misused or abused prescription opioids, resulting in adverse health effects or even accidental death [2-4]. According to the Health Resources and Services Administration, more than 130 people die from OUDs daily [2]. Researchers attribute the rise of OUD as a national epidemic to increased prescribing of opioids in the 1990s, with overdose deaths (involving prescription opioids, natural and semisynthetic opioids, and methadone) increasing since at least 1999 [5]. The opioid crisis and subsequent increase in OUD are cited as beginning when the shift in prescribing opioid-derived medications was used for more common forms of pain rather than the palliative care for which it was first intended [6].

Several preexisting health issues and behavioral risk factors were shown to be associated with a higher likelihood of OUD. For instance, individuals with mood and anxiety disorders were subject to experiencing chronic pain, leading to increased use of opioid-based drugs prescribed by physicians [7-9]. In fact, preexisting diagnoses of mood and anxiety disorders increased the risk of opioid prescription by 50% [10,11]. Some other psychosocial and psychiatric disturbances, including cultural influences, social support, comorbid mood disorder, and drug abuse, were found to be associated with higher levels of chronic pain, which could significantly enhance the risk of opioid overuse [12,13]. Additionally, there are some behavioral risk factors for OUD, such as chronic substance use, current or past substance abuse, family history of substance abuse, overconsumption of alcohol, posttraumatic stress disorder, and physical abuse [14-17].

Opioid-related morbidity and mortality are highly prevalent in workplaces in the United States. In particular, health care, construction and extraction, transportation, and warehouse workers are more vulnerable. In these industries, where major activities and responsibilities are physically and mentally demanding, many workers are susceptible to physical and mental health issues such as chronic pain and mental disorders leading to opioid overuse or misuse [18,19]. Epidemiological occupational health studies indicated that high physical and stressful job demands were strongly associated with OUD [18,20-23]. Therefore, combining on-the-job risk factors for injury coupled with provider prescription recommendations for opioid use could elevate the risk of developing OUD within these occupational groups [1].

To mitigate the risk factors and reduce opioid overuse among vulnerable workforces, there is a critical need to develop evidence-based interventions to reduce the magnitude of OUD and remove the psychosocial and workplace barriers to effective interventions. Current behavioral interventions for OUD include cognitive behavioral therapy [24], group therapy sessions [24], questionnaires or evaluations [24,25], manuals [24], motivational interviewing [26], and mobile phone apps offering support during recovery [27]. Some proven intervention approaches include educating providers, persons at risk, and their family members about how to prevent opioid overdose; ensuring access to treatment for the affected individuals; ensuring access to naloxone, an appropriate response to opioid overdose events; and encouraging prescribers to use state prescription drug monitoring programs [28-30]. However, most of these approaches require large-scale coordination and strong health care infrastructure and often involve expensive intervention strategies. Mobile health (mHealth) interventions may be considered promising and low-cost tools to address this challenge in a robust yet cost-effective manner.

We have developed a multifeature smartphone app targeting 2 critical occupational groups in Texas—the transportation and health care workforce. The primary goal is to reduce the prevalence of opioid overuse in these critical infrastructure sectors and promote the health and well-being of the employees via technology-based intervention. We believe that our innovative educational public health technology will enhance the security and resilience in these 2 industries, allowing them to continue their business in a healthier and more productive environment. This concept paper examines the risk factors of opioid use among vulnerable labor industries, including transportation and health care workers, in Texas through a quick and easy-to-use screening approach and proposes potential technology, a smartphone app, for assessing and mitigating OUD. Our study illustrates the method of app development, experiments to evaluate the app, and future directions for its use.

## Methods

### Approach and Procedures

Health interventions that have depended solely on face-to-face delivery can now be widely used outside the clinical settings via mHealth tools. This research indicates that smartphone apps are easily accessible and allow patients to access the required information upon request whenever necessary [31,32]. The main advantages of a smartphone app are to offer multimedia features that combine texts and audiovisual information, comprehensive activation, reproducibility, and final feedback about a health issue [32,33]. However, the limitation of this study is that it used synthetic data for the pilot study, and no human participant data was involved. Additionally, the effectiveness of the app on the target population needs to be validated through a field study.

### Development and Design of the Smartphone App

We developed a cross-platform smartphone app for both iOS and Android users. The app can keep track of work-related risk factors and determine the risk of OUD based on the individual responses. We select several questionnaires from the extensive literature review, and the smartphone app user will respond to those. All responses in the app will be collected and used as an input for the machine learning model to predict the risk of OUD. However, all responses are stored in a cloud-based database that allows for multilayer security via user validation and data encryption. Protecting data from unauthorized access is essential because of participants’ sensitive medical information.

The proposed app was built with the Flutter framework for cross-platform mobile apps. Flutter is an open-source framework by Google for building user-friendly, natively compiled multiplatform applications from a single codebase. The agile development method is used to develop the smartphone app [34]. Software development occurred in 4 cycles. For each cycle, the first step is determining and analyzing the project requirement. Next, the smartphone app is designed with the research team’s input and vision. The research team builds and tests small prototypes of the app to identify flaws. With the feedback, smartphone app adjustments are made accordingly, and the process of analysis, design, coding, and building a prototype is repeated.

**Figure 1:**
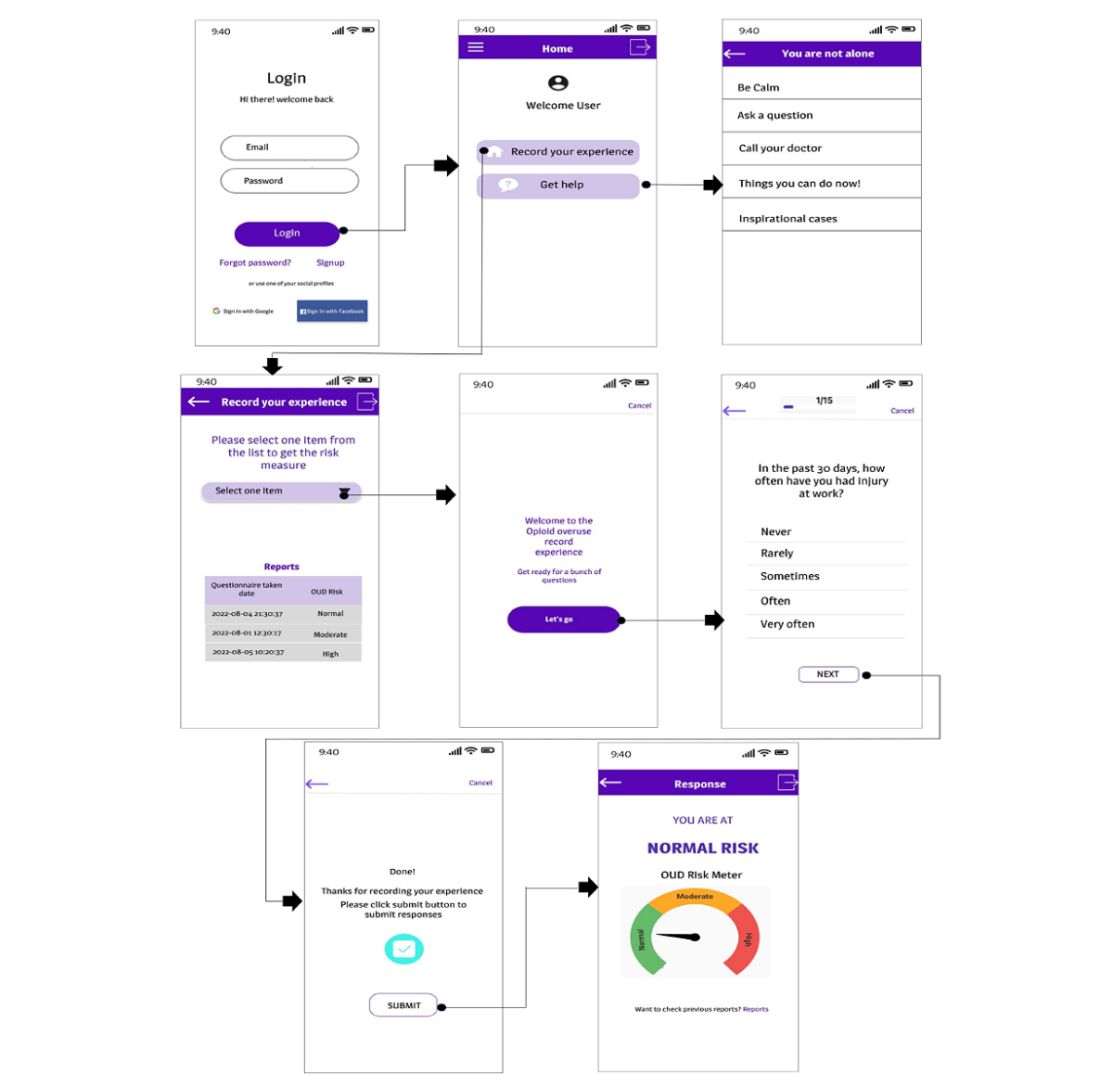
The wireframe of the developed mobile app, a simplified description. OUD: opioid use disorder.

In addition, we use Firebase as a cloud-based database, a platform Google developed for creating mobile and web apps. A simplified wireframe of the developed multifeatured smartphone app is described in Figure 1.

### Features and Navigation of the Mobile App

**Figure 2:**
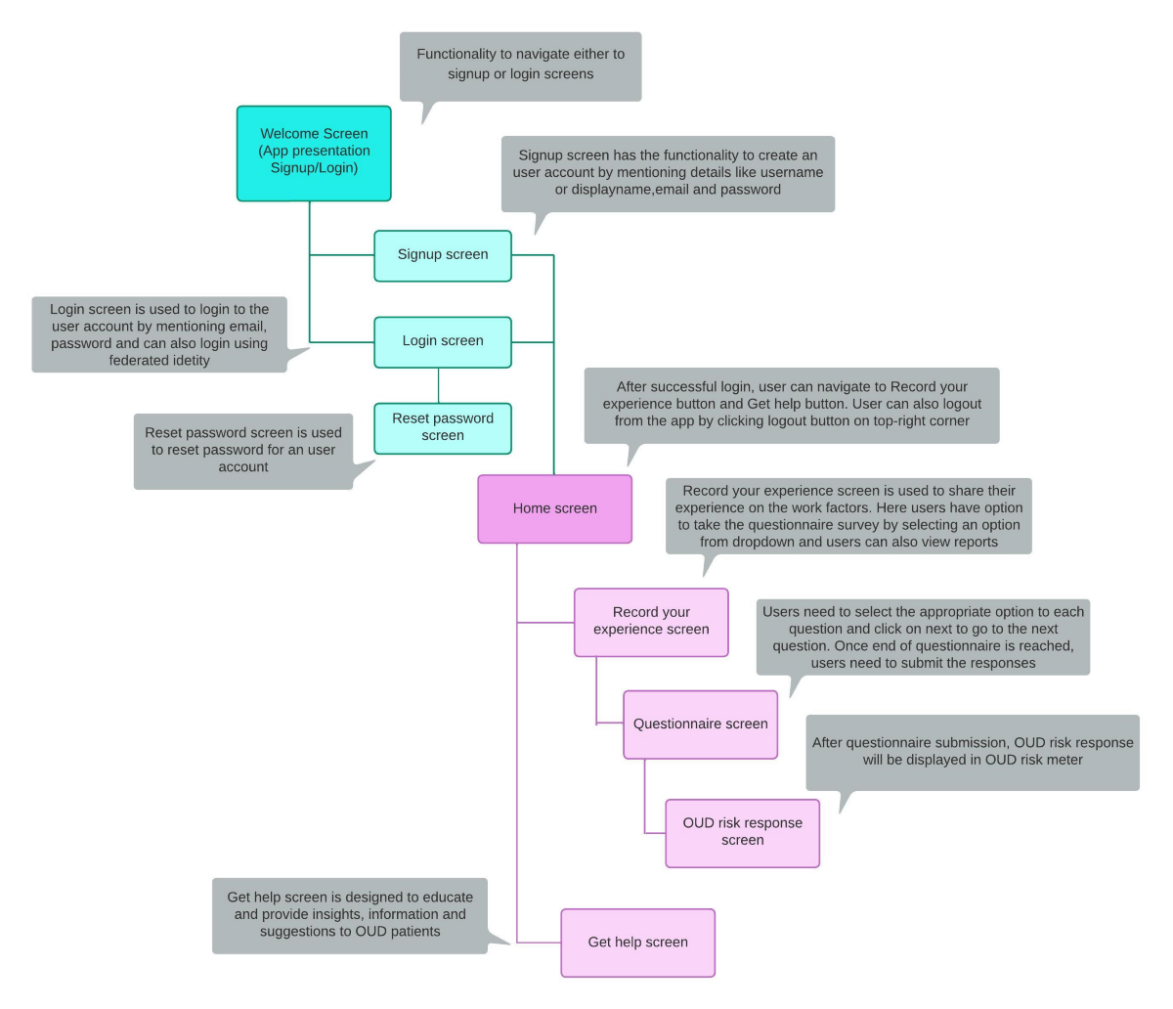
App screens and their sequence and functionality. OUD: opioid use disorder.

The mobile app has several pages, and each page has related functionality. Figure 2 shows app screens and their sequence and functionality.

### Procedure

The following procedure will be used:

1. Sign up or log in: Users can create an account by using their name, email address, and password. Once the signup process is over, users can log into the account. Users can also use federated identity by login using Google, Facebook, or Apple accounts.
2. Users must navigate to record their experience with opioid use on successful login.
3. Users need to give responses to the questionnaire and submit the responses.
4. On successful submission, a machine learning algorithm (naive Bayes [NB]) will analyze the submitted responses and give an OUD risk response.
5. Users can also view the previously submitted questionnaire OUD risk responses in a tabular format on the “Record your experience” page.
6. Users can navigate to the get help page to get insights, information, and educational intervention for patients with OUD.

### Feature Extraction and Processing

An extensive literature review was conducted to identify the risk factors contributing to opioid overuse. A pilot questionnaire consisting of 15 questions has been developed to collect risk factor data. We followed a 3-step process while developing the OUD assessment questionnaire. In the first step, we selected articles containing opioid overuse–related questions that received at least 5 citations. Next, we identified the frequencies of different questions across the selected articles and questions; the questions that had the highest frequency were eventually selected for the project. Finally, our 6-member resource panel evaluated the questions and made small revisions whenever necessary. These questions are classified into 3 categories: 6 of them were highly sensitive, 4 were moderately sensitive, and 5 were mildly sensitive questions. Of the 15 questions, 9 have two response options (yes or no), 5 have five response options, and 1 has three response options. However, all the responses need to convert to numerical values for further processing. Our ultimate goal is to provide a response to the user about OUD based on the response given. A machine learning model NB was used to predict the OUD risk level. All responses were given weights or scores. We have used Likert scales to develop the scoring system, a widely used method in the field of public health. A Likert scale is a validated method to assess attitude and behavior [35,36]. An analysis of various scoring patterns for the risk factors and weights or scores was done on a Likert-type scale. The scoring pattern for the question responses is given in Tables 1-3.

A data set using risk factors and scores was developed. The data set combines various responses and is used as an input for an NB classifier model. Among all machine learning algorithms, NB is used here in this project because it is suitable for binary and multiclass classification. NB works well in cases of categorical input variables compared with numerical variables. NB is useful for making predictions based on past results. Moreover, it is simple. If the conditional independence assumption holds, an NB classifier will converge quicker than discriminative models like logistic regression, so we need less training data. Moreover, even if the NB assumption does not hold, it requires less model training time. The NB model will predict 3 categories: normal, moderate, and high risk of OUD, for the given test data.

**Table 1:** Scoring for question responses based on 5 option questions.

Question response	Scoring
Never	1.8
Rarely	2.6
Sometimes	3.4
Often	4.2
Very often	5.0

**Table 2:** Scoring for question responses for 3 option questions.

Question response	Scoring
Mild	1.8
Moderate	3.4
Severe	5.0

**Table 3:** Scoring for question responses for 2 option questions.

Question response	Scoring
Yes	5.0
No	1.8

### Machine Learning Model

This project implements a supervised machine learning model called the NB classifier. The NB classifier is simple and fast, provides satisfactory results for classification tasks, and is easy to implement. NB methods are a set of supervised machine learning algorithms based on applying Bayes’ theorem and using the “naive” assumption that features are independent of each other [1]. Bayes’ theorem states the following relationship between the class variable “*y*” and feature vector “*x*_1_” through “*x_n_*”:



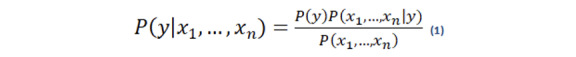



If we modify all features from equation 1 using “*i*,” the equation can be simplified to:



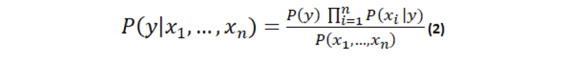



which is the probability for a class variable and can be simplified for more class variables to:



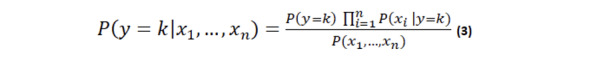



which is the probability for each class variable.

We compute the probability of each class of “*y*” and choose the class with the highest probability as the prediction. NB learners and classifiers can be extremely fast compared with more sophisticated methods. This is one of the reasons to choose the NB algorithm since the app will run on a smartphone, and the app must have a quick response time as a usability feature. The NB algorithm was developed as CategoricalNB by the scikit-learn library [2]. CategoricalNB is one type of algorithm of the NB classifier for categorically distributed data. It assumes that each feature described by the index has its own categorical distribution. For each feature “*i*” in the training set, CategoricalNB estimates a categorical distribution for each feature “*i*” of *X* conditioned on the class “*y.*” The index set of the samples is defined as *J*={1,…,*m*}, with “*m*” the number of samples.

The probability of category “*t*” in the feature “*i*” given class “*c*” is estimated as:



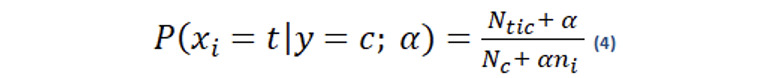



where 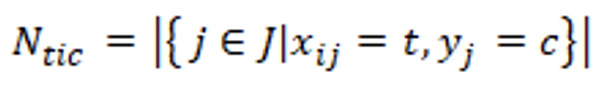 is the number of times category *t* appears in the samples “*x_i_*,” which belong to the class “*c_i_*.” 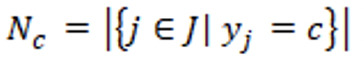 is the number of samples with class *c*, *α* is a smoothing parameter, and *n_i_* is the number of available categories of feature *i.*

The pseudocode for the NB classifier is described in Textbox 1.

**Pseudocode of the naive Bayes classifier.**

Procedure naive Bayes classifier (*X*)

{

Compute the “Prior” probabilities for each of the classes of *Y*

*P* (*Y_i_*)

Compute the probability of likelihood feature vectors for each target vector

*P* (*X*|*Y_i_*)

Compute the probability of feature vectors, that is, the product of probabilities of *X*’s for all *X*

*P*( *X*_1_,…, *X_n_*)

Calculate probabilities of all the classes of *Y* by using

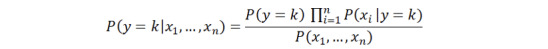

Choosing the class *Y* with the highest probability as the predicted class

}

### Machine Learning Model Implementation

We have implemented the machine learning model using the scikit-learn library [2], a free software machine learning library using Python programming. We have used the CategoricalNB algorithm, which implements the NB classifier for categorical features by the scikit-learn library [3] and implements the model on the application powered by Google Colaboratory (Google Colab) TPU with a high-RAM environment, an entirely cloud-based Jupyter Notebook environment. The data set created during feature extraction is used for training the machine learning model. Once the training of the machine learning model is completed, we use the pickle operation to serialize the machine learning algorithm. We create a representational state transfer (REST) application programming interface (API) using Flask, a Python framework used as the back end. We host this REST API onto Heroku, a platform as a service that enables developers to build, run, and operate applications entirely in the cloud. Heroku runs the app in lightweight, isolated Linux containers called “dynos.” We use the free tier for our application. In the REST API, we deserialize the machine learning algorithm back to the Python object and predict the OUD risk responses with input data sent from the mobile app. We send requests to this REST API and questionnaire responses submitted from the mobile app. The predicted OUD risk response will be sent back to the mobile app. The returned response from REST API will be displayed on the Response page. The response from the machine learning model will determine the risk factor of OUD for an individual, whether a respondent is at normal, moderate, or high risk of OUD.

### Data Sets

Data have been captured from users for 15 questions, considering the 15 responses as different features for the machine learning model. As we do not have human participants involved in our research, we have created a data set by analyzing the literature review. We have classified these 15 questions into 3 different categories. Six questions are classified as highly sensitive questions, which means there is a high risk of opioid overuse; 4 questions are classified as moderately sensitive, which means there is a moderate risk of opioid overuse. Furthermore, 5 questions are classified as mild sensitive, meaning the low risk of opioid overuse. This data set consists of 15 feature vectors and 1 target vector, which will be the output. Our target vector consists of 3 classes, that is, normal, moderate, and high risk. We have assigned these 3 classes to 3 numerical values (normal=1, moderate=2, and high=3). Each feature vector is assigned a weight or score based on the Likert scale, as mentioned in the Feature Extraction and Processing section. We have created a training data set consisting of 23 samples with 15 feature vectors and a target vector. This data set is used for training the machine learning model.

We have prepared a data set using Likert scale weights, and all the questions or features are considered with equal weightage. We have created a data set by considering weightage to the features based on the category of questions, that is, considering more weightage to highly sensitive questions and less weightage to mild sensitive questions. In order to add weightage to each category of questions, we have considered 50% of the score of highly sensitive questions, 30% of the score of moderately sensitive questions, and 20% of the score of mildly sensitive questions.

### Ethical Considerations

As this study did not involve human participants, there were no ethical concerns related to confidentiality, informed consent, or other potential risks associated with research involving human participants. Instead, we used synthetic data generated through simulation methods to investigate the research question. Using synthetic data ensured that there were no privacy concerns or risks associated with identifying individual participants. Therefore, no special ethical considerations were necessary for this study.

### Experiment

**Figure 3:**
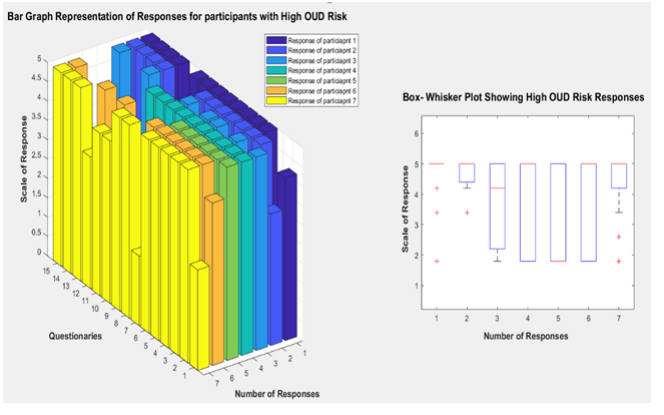
Bar graph and box-whisker plot depicting high OUD risk response. OUD: opioid use disorder.

**Figure 4:**
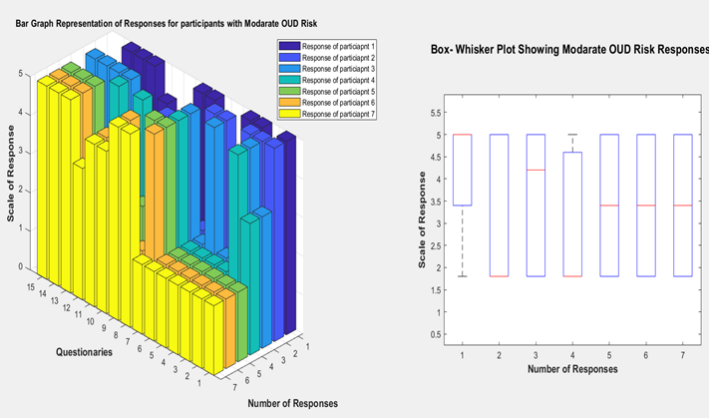
Bar graph and box-whisker plot depicting moderate OUD risk response. OUD: opioid use disorder.

**Figure 5:**
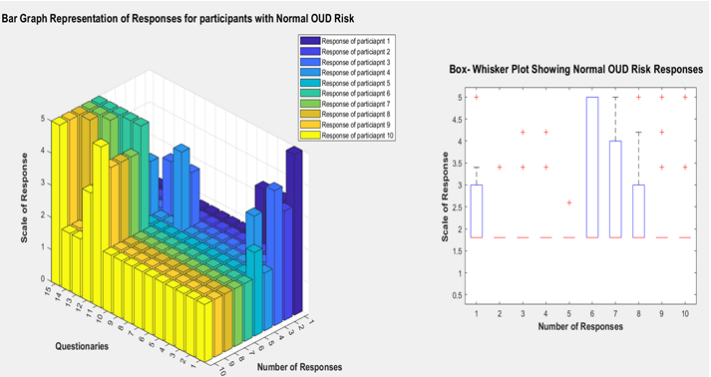
Bar graph and box-whisker plot depicting normal OUD risk response. OUD: opioid use disorder.

A research experiment using a developed mobile app was conducted. The machine learning model has been implemented using an application powered by the Google Colab environment and trained using a created training data set. Once training had been completed, we deployed this model on the Heroku environment and accessed this trained model using REST API from the developed mobile app. We use Firebase as our database for a mobile app that Google is developing. Firebase also offers other services, including authentication, analytics, and so on. Once the user signs up using the required details, the home screen opens, where the user can navigate to the questionnaire screen. The user enters a response to all the questions and submits the responses. Then the machine learning algorithm calculates probabilities for each class of target vector and outputs the class with the highest probability as a prediction. The predicted OUD risk factor will be displayed on the mobile app response screen. Users can also check previously recorded OUD risk factor data in the mobile app. Figures 3-5 show graphs depicting the feature vector relationship with OUD risk response.

## Results

We have generated synthetic data to evaluate the proposed machine learning algorithm for experimental purposes. Our domain expert labeled the responses with the risk factors, and we validated that with the machine learning algorithm. Figure 3 describes the response of participants for the developed questionnaires at high risk of OUD. In the bar graph, the x-axis represents the number of participants, which is 7; in this case, the y-axis is the 15 questionnaires we developed from an extensive literature search. The z-axis is the score of the responses. In the bar graph of Figure 3, most participants responded high (on the scale of responses) for the first 6 questions. It is worth mentioning that the questions are ordered from high to low risk. That means if any participant responds higher scale in questions 1-6, they are most likely to be at a high risk of OUD. Compared with the moderate and normal OUD risk described by the bar chart in Figures 4 and 5, the participants responded higher in the mid (7-10) and low (10-15) part of the survey questionnaires order.

In the box and whisker plot, the central mark indicates each box’s median, and the box’s bottom and top edges indicate the 25th and 75th percentiles, respectively. The whiskers extend to the most extreme data points not considered outliers, and the outliers are plotted individually using the “+” symbol. In Figure 3, the box and whisker plot indicates that the participants with a high risk of OUD have a median response of around 5, which means “very often” or “yes.” The median for the moderate-risk participants is around 3.5, meaning they mostly respond with “sometimes” or “rarely.” This trend also holds for the participants with low risk; the median of their responses is around 1.75, indicating that their responses are usually “never,” “rarely,” or “no.”

## Discussion

### Main Findings

OUD is a disease of epidemic proportions in the United States [1]. Studies have indicated that the opioid epidemic has significantly strained the American economy [37,38]. The strains can be felt through the insurmountable health care costs, the strain on the judicial and criminal system, and the accrued loss of productivity [39-41]. The overall outcome of these crises is a deficit of indirect costs associated with increased morbidity and mortality for specific occupations, thus creating a decline in productivity and increasing high costs [41].

Furthermore, individuals employed in areas that have a higher than usual occupational strain—either physical or mental—have been noted to have high premature mortality and loss of productivity, which create an economic burden. Also, occupational injuries because of the nature of the employment result in employees using higher amounts of opioid-based medications [41].

Our study aimed at creating an app used by individuals in high-risk occupations—transportation and health care—to assess individual risk for OUD. An extensive literature review indicated that occupations in which workers are repeatedly exposed to stress and occupations that are physically demanding result in overprescription of opioid-based drugs [18,19]. The study design incorporated mHealth technologies in creating a smartphone app so that individuals in those occupations may confidentially assess their risk level for OUD in privacy at their residence as the traditional questionnaires or inquiries by physicians prove unreliable due to the sensitive nature of the disease. Using an app that can be accessed in privacy ensures an increase in validity [42]. After individuals take the assessment, they can make notes within the app for their provider and are offered resources for getting help. We have created a private pathway for assessing risk and aiding treatment by implementing this technology.

### Strengths and Limitations

The project has many strengths that offer support in its use. First, the app has been designed for both iOS and Android operating systems, ensuring that individuals can access the app regardless of their preferred operating system. The app is user-friendly and consists of 15 questions. This feature was created after extensively reviewing the literature to determine risk factors and behaviors highly correlated with OUD. The questions are categorized according to high-, medium-, and low-risk factors ensuring precision and the risk analysis for OUD at an individual level. Furthermore, studies indicate that health care apps are highly effective in shaping health attitudes, empowering patients, and “pushing” them in the direction of managing their condition appropriately [43-45]. This ultimately lends itself to the app having a precise related functionality.

The app’s most highlighted strength is that it uses an NB model in statistically analyzing the 15 questions individually. The NB model has been widely used for classifying and clustering outcomes, but this project uses the model and probabilistic interface [46]. Machine learning models, such as NB, have been used exclusively for data mining. However, the current trend is to use them in disease prevention [47]. The app also uses a REST API, thus further ensuring the confidentiality of the patient.

As with all apps, there are some limitations. Self-administered instruments run the risk in that the respondents do not answer truthfully or minimize their responses to ensure a desirable outcome or appearance of the responses [48,49]. Currently, the app has undergone several pretesting phases to assess the accuracy of analyzing the respondents’ responses. We are still in the process of working with Apple to launch the app commercially. As a result, we cannot guarantee that the graphical user interface is navigable for our target audience [50,51].

### Future Goals

The future goals of this project are to expand the app’s functionality and include some educational intervention features. The proposed app will be used as a launching pad for various mHealth-based assessments. Moreover, we used an artificial intelligence–based algorithm, and as the number of users increases, the algorithm can predict better risk factors. After conducting several tests or pilot studies of the app, we can administer this app to the communities identified as high-risk, such as health care, construction and extraction, transportation, and warehouse industries.

### Conclusions

The current trend indicates that use of mHealth techniques, such as a mobile app, is favorable in predicting and offering mitigation plans for disease detection and prevention [47,52-55]. Amidst the ever-increasing opioid epidemic, occupations of note, such as transportation and construction, have encountered alarming issues related to opioids. Because of the intense physical and mental strain and stress placed on employees in these fields, studies indicate that they face a higher risk of developing OUD [18,19].

Recognizing the current movement to use mHealth technologies and the sensitive nature of self-reporting one’s level of risk for developing OUD, the development of our smartphone app offers a promising tool for addressing the opioid crisis in specific workforces. The use of artificial intelligence in predicting an individual’s OUD risk offers a novel approach to prevention and intervention. The implementation of mHealth tools, such as our app, has the potential to provide convenient and effective interventions outside of traditional clinical settings. We hope that the development of this app will contribute to a reduction in opioid misuse and provide tailored mitigation strategies to communities most impacted by OUD. Further research is needed to validate the effectiveness of our app and to ensure that it meets the needs of the target population.

## Abbreviations

API: application programming interface
mHealth: mobile health
NB: naive Bayes
OUD: opioid use disorder
REST: representational state transfer

## References

[1] Shaw WS, Roelofs C, Punnett L (2020). Work environment factors and prevention of opioid-related deaths. Am J Public Health.

[2] (2020). Opioid crisis. Health Resources and Services Administration.

[3] Scholl L, Seth P, Kariisa M, Wilson N, Baldwin G (2018). Drug and Opioid-Involved Overdose Deaths - United States, 2013-2017. MMWR Morb Mortal Wkly Rep.

[4] Stringfellow EJ, Lim TY, Humphreys K, DiGennaro C, Stafford C, Beaulieu E, Homer J, Wakeland W, Bearnot B, McHugh RK, Kelly J, Glos L, Eggers SL, Kazemi R, Jalali MS (2022). Reducing opioid use disorder and overdose deaths in the United States: a dynamic modeling analysis. Sci Adv.

[5] Manchikanti L, Singh VM, Staats PS, Trescot AM, Prunskis J, Knezevic NN, Soin A, Kaye AD, Atluri S, Boswell MV, Abd-Elsayed A, Hirsch JA (2022). Fourth wave of opioid (illicit drug) overdose deaths and diminishing access to prescription opioids and interventional techniques: cause and effect. Pain Physician.

[6] Volkow ND, Jones EB, Einstein EB, Wargo EM (2019). Prevention and treatment of opioid misuse and addiction: a review. JAMA Psychiatry.

[7] Sheng J, Liu S, Wang Y, Cui R, Zhang X (2017). The link between depression and chronic pain: neural mechanisms in the brain. Neural Plast.

[8] Arango-Dávila CA, Rincón-Hoyos HG (2018). Depressive disorder, anxiety disorder and chronic pain: multiple manifestations of a common clinical and pathophysiological core. Rev Colomb Psiquiatr (Engl Ed).

[9] Humo M, Lu H, Yalcin I (2019). The molecular neurobiology of chronic pain-induced depression. Cell Tissue Res.

[10] Goesling J, Henry MJ, Moser SE, Rastogi M, Hassett AL, Clauw DJ, Brummett CM (2015). Symptoms of depression are associated with opioid use regardless of pain severity and physical functioning among treatment-seeking patients with chronic pain. J Pain.

[11] Halbert BT, Davis RB, Wee CC (2016). Disproportionate longer-term opioid use among U.S. adults with mood disorders. Pain.

[12] Gatchel RJ, Peng YB, Peters ML, Fuchs PN, Turk DC (2007). The biopsychosocial approach to chronic pain: scientific advances and future directions. Psychol Bull.

[13] Rosenblum A, Marsch LA, Joseph H, Portenoy RK (2008). Opioids and the treatment of chronic pain: controversies, current status, and future directions. Exp Clin Psychopharmacol.

[14] Leung K, Xu E, Rosic T, Worster A, Thabane L, Samaan Z (2021). Sensitivity and specificity of self-reported psychiatric diagnoses amongst patients treated for opioid use disorder. BMC Psychiatry.

[15] Zhu Y, Mooney LJ, Yoo C, Evans EA, Kelleghan A, Saxon AJ, Curtis ME, Hser YI (2021). Psychiatric comorbidity and treatment outcomes in patients with opioid use disorder: results from a multisite trial of buprenorphine-naloxone and methadone. Drug Alcohol Depend.

[16] Ober AJ, Hunter SB, McCullough CM, Leamon I, McCreary M, Beas I, Montero A, Tarn DM, Bromley E, Hurley B, Sheehe J, Martinez J, Watkins KE (2022). Opioid use disorder among clients of community mental health clinics: prevalence, characteristics, and treatment willingness. Psychiatr Serv.

[17] (2022). Opioid use disorder. Yale Medicine.

[18] Choi B (2020). Opioid use disorder, job strain, and high physical job demands in US workers. Int Arch Occup Environ Health.

[19] Dale AM, Buckner-Petty S, Evanoff BA, Gage BF (2021). Predictors of long-term opioid use and opioid use disorder among construction workers: analysis of claims data. Am J Ind Med.

[20] Karasek RA (1979). Job demands, job decision latitude, and mental strain: implications for job redesign. Adm Sci Q.

[21] Davey J, Richards N, Freeman J (2007). Fatigue and beyond: patterns of and motivations for illicit drug use among long-haul truck drivers. Traffic Inj Prev.

[22] Barcenilla A, March LM, Chen JS, Sambrook PN (2012). Carpal tunnel syndrome and its relationship to occupation: a meta-analysis. Rheumatology (Oxford).

[23] Walter AW, Morocho C, King L, Bartlett J, Kelsey D, DeSousa M, Biesecker G, Punnett L (2018). Preventing opioid use disorders among fishing industry workers. Int J Environ Res Public Health.

[24] Mumba MN, Mugoya GT, Smith NL, Glenn A, Potts C, Campbell MH, Kirwan C, Butler A, Davis L (2020). Development of a novel behavioral intervention for opioid use disorders. Online J Issues Nurs.

[25] Brown KG, Capili B (2020). CE: opioid use disorder: pathophysiology, assessment, and effective interventions. Am J Nurs.

[26] Compton P, Darakjian J, Miotto K (1998). Screening for addiction in patients with chronic pain and "problematic" substance use: evaluation of a pilot assessment tool. J Pain Symptom Manage.

[27] Hochstatter KR, Gustafson DH, Landucci G, Pe-Romashko K, Cody O, Maus A, Shah DV, Westergaard RP (2021). Effect of an mHealth intervention on hepatitis C testing uptake among people with opioid use disorder: randomized controlled trial. JMIR Mhealth Uhealth.

[28] Reisman RM, Shenoy PJ, Atherly AJ, Flowers CR (2009). Prescription opioid usage and abuse relationships: an evaluation of state prescription drug monitoring program efficacy. Subst Abuse.

[29] Patrick SW, Fry CE, Jones TF, Buntin MB (2016). Implementation of prescription drug monitoring programs associated with reductions in opioid-related death rates. Health Aff.

[30] Bao Y, Pan Y, Taylor A, Radakrishnan S, Luo F, Pincus HA, Schackman BR (2016). Prescription drug monitoring programs are associated with sustained reductions in opioid prescribing by physicians. Health Aff.

[31] Rathbone AL, Prescott J (2017). The use of mobile apps and SMS messaging as physical and mental health interventions: systematic review. J Med Internet Res.

[32] Sheikh A, Anderson M, Albala S, Casadei B, Franklin BD, Richards M, Taylor D, Tibble H, Mossialos E (2021). Health information technology and digital innovation for national learning health and care systems. Lancet Digit Health.

[33] Malale K, Fu J, Nelson W, Gemuhay HM, Gan X, Mei Z (2020). Potential benefits of multimedia-based home catheter management education in patients with peripherally inserted central catheters: systematic review. J Med Internet Res.

[34] Paulk MC (2002). Agile methodologies and process discipline. CrossTalk: J Defense Softw Eng.

[35] Nuamah JK, Sasangohar F, Erraguntla M, Mehta RK (2019). The past, present and future of opioid withdrawal assessment: a scoping review of scales and technologies. BMC Med Inform Decis Mak.

[36] Giblin MJ, Cordaro M, Haskard-Zolnierek K, Jordan K, Bitney C, Howard K (2022). Identifying the risk of opioid misuse in a chronic pain population: the utility of the MMPI-2-RF personality psychopathology five (PSY-5-RF) and higher-order scales. J Behav Med.

[37] Birnbaum HG, White AG, Schiller M, Waldman T, Cleveland JM, Roland CL (2011). Societal costs of prescription opioid abuse, dependence, and misuse in the United States. Pain Med.

[38] Rice JB, Kirson NY, Shei A, Enloe CJ, Cummings AK, Birnbaum HG, Holly P, Ben-Joseph R (2014). The economic burden of diagnosed opioid abuse among commercially insured individuals. Postgrad Med.

[39] Florence C, Luo F, Rice K (2021). The economic burden of opioid use disorder and fatal opioid overdose in the United States, 2017. Drug Alcohol Depend.

[40] Harwood HJ, Fountain D, Livermore G, Lewin Group, National Institute on Drug Abuse, National Institute on Alcohol Abuse and Alcoholism (U.S.) (1998). The Economic Costs of Alcohol and Drug Abuse in the United States, 1992.

[41] Grosse SD, Krueger KV, Pike J (2019). Estimated annual and lifetime labor productivity in the United States, 2016: implications for economic evaluations. J Med Econ.

[42] Ng MM, Firth J, Minen M, Torous J (2019). User engagement in mental health apps: a review of measurement, reporting, and validity. Psychiatr Serv.

[43] Mano RS (2014). Social media and online health services: a health empowerment perspective to online health information. Comput Hum Behav.

[44] Luxton DD, McCann RA, Bush NE, Mishkind MC, Reger GM (2011). mHealth for mental health: integrating smartphone technology in behavioral healthcare. Prof Psychol Res Pr.

[45] Mano R (2021). Mobile health apps and health management behaviors: cost-benefit modeling analysis. JMIR Hum Factors.

[46] Lowd D, Rooshenas A (2015). The libra toolkit for probabilistic models. J Mach Learn Res.

[47] Uddin S, Khan A, Hossain ME, Moni MA (2019). Comparing different supervised machine learning algorithms for disease prediction. BMC Med Inform Decis Mak.

[48] Collen MF, Cutler JL, Siegelaub AB, Cella RL (1969). Reliability of a self-administered medical questionnaire. Arch Intern Med.

[49] Turconi G, Celsa M, Rezzani C, Biino G, Sartirana MA, Roggi C (2003). Reliability of a dietary questionnaire on food habits, eating behaviour and nutritional knowledge of adolescents. Eur J Clin Nutr.

[50] Shneiderman B, Plaisant C, Cohen M, Jacobs S, Elmqvist N, Diakopoulos N (2016). Designing the User Interface: Strategies for Effective Human-Computer Interaction.

[51] Norman KL, Friedman Z, Norman K, Stevenson R (2001). Navigational issues in the design of online self-administered questionnaires. Behav Inf Technol.

[52] Ray R, Sewell AA, Gilbert KL, Roberts JD (2017). Missed opportunity? Leveraging mobile technology to reduce racial health disparities. J Health Polit Policy Law.

[53] Ben-Zeev D, Schueller SM, Begale M, Duffecy J, Kane JM, Mohr DC (2015). Strategies for mHealth research: lessons from 3 mobile intervention studies. Adm Policy Ment Health.

[54] Davis DA, Chawla NV, Christakis NA, Barabási AL (2010). Time to CARE: a collaborative engine for practical disease prediction. Data Min Knowl Disc.

[55] McCormick T, Rudin C, Madigan D (2011). A hierarchical model for association rule mining of sequential events: an approach to automated medical symptom prediction. SSRN J.

